# Supplementary figures and images for: Transcriptome Analysis Reveals Mechanisms of Stripe Rust Response in Wheat Cultivar Anmai1350
Source: Int J Mol Sci. 2025 Jun 10;26(12):5538. doi: 10.3390/ijms26125538 (PMC12192900; doi:10.3390/ijms26125538)

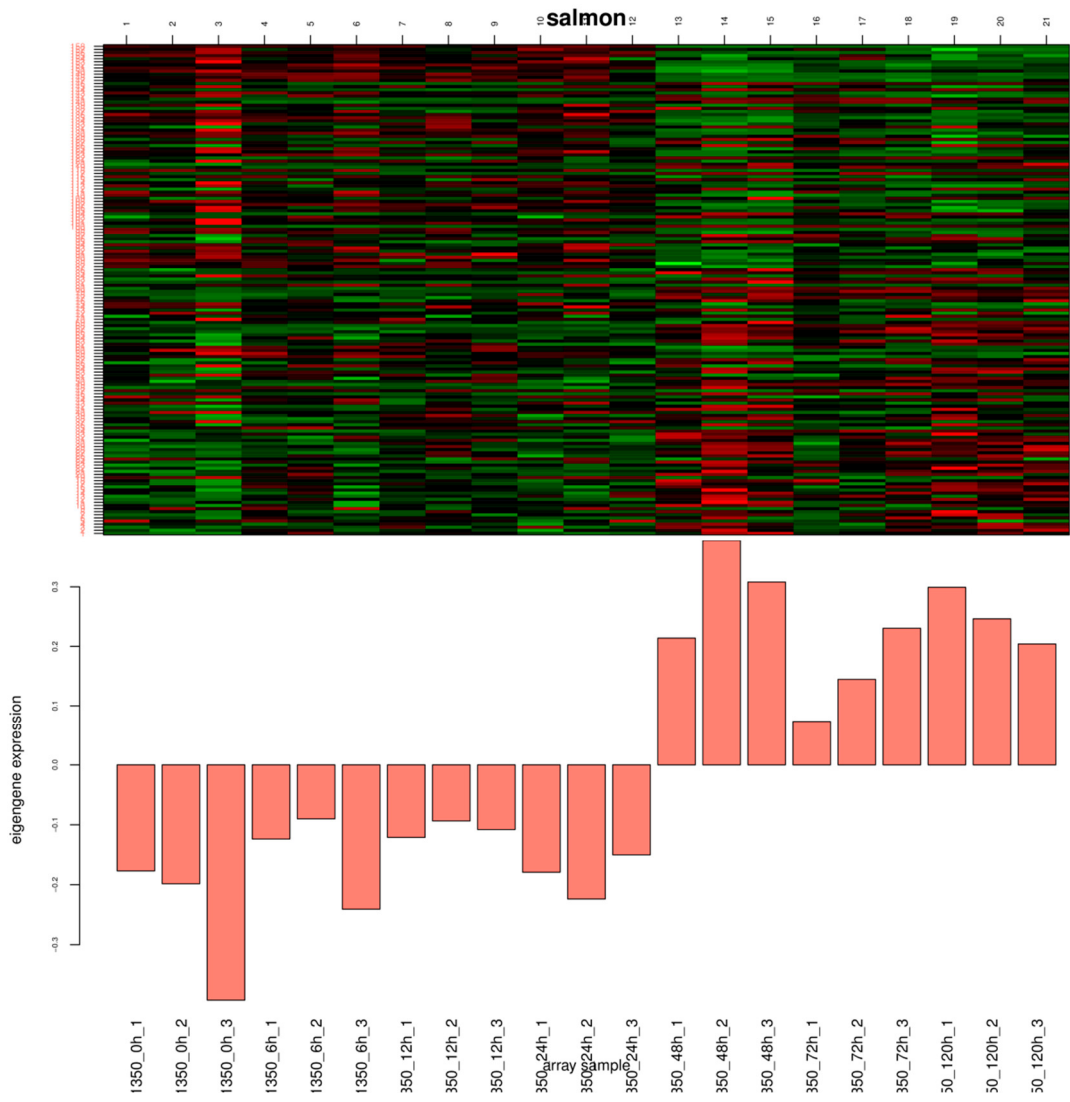

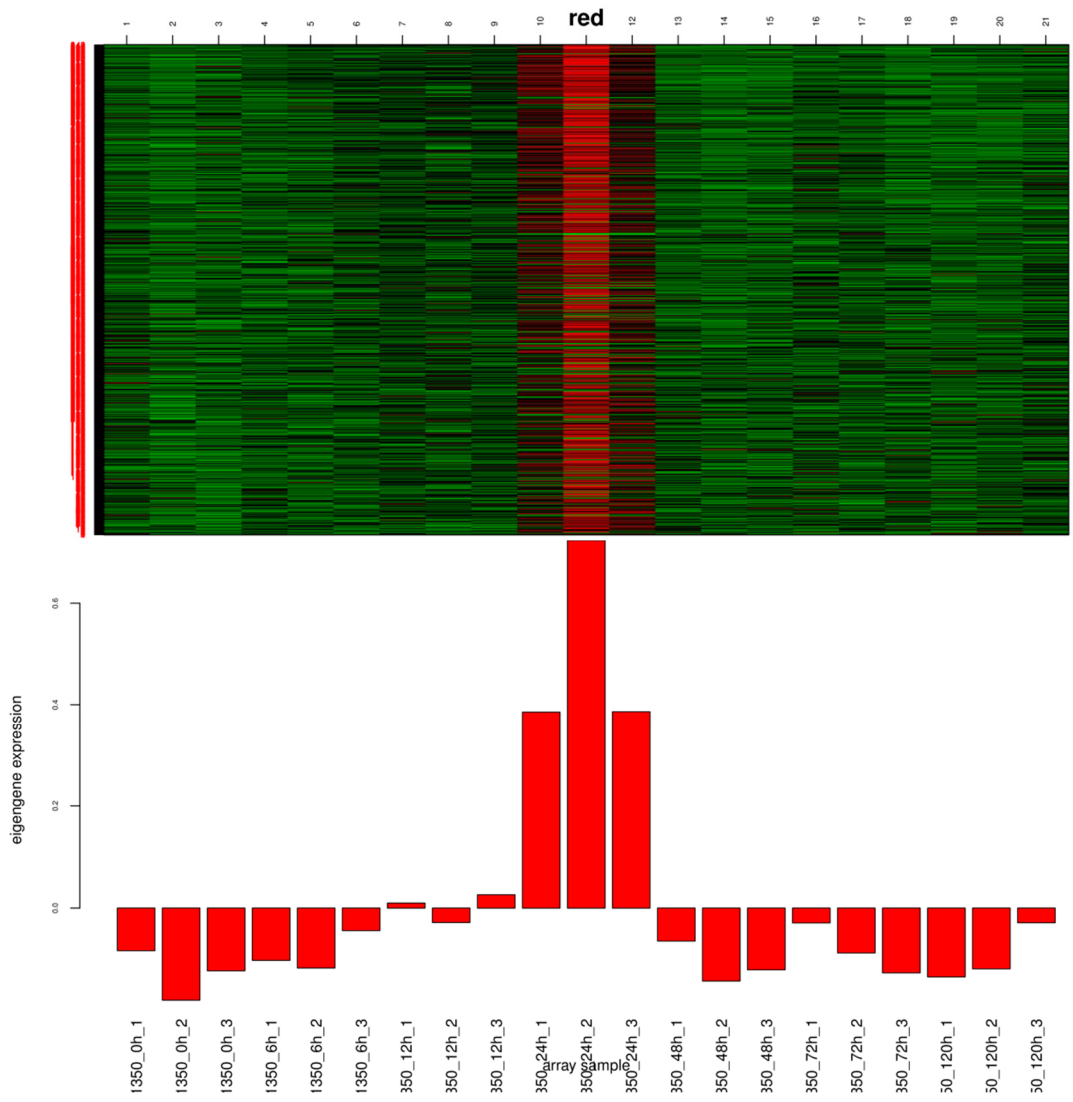

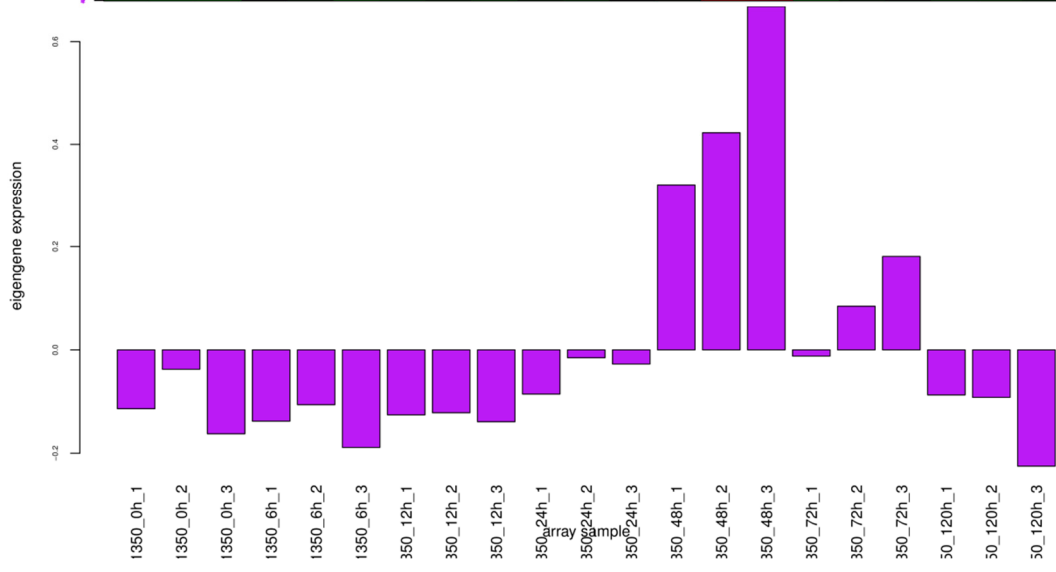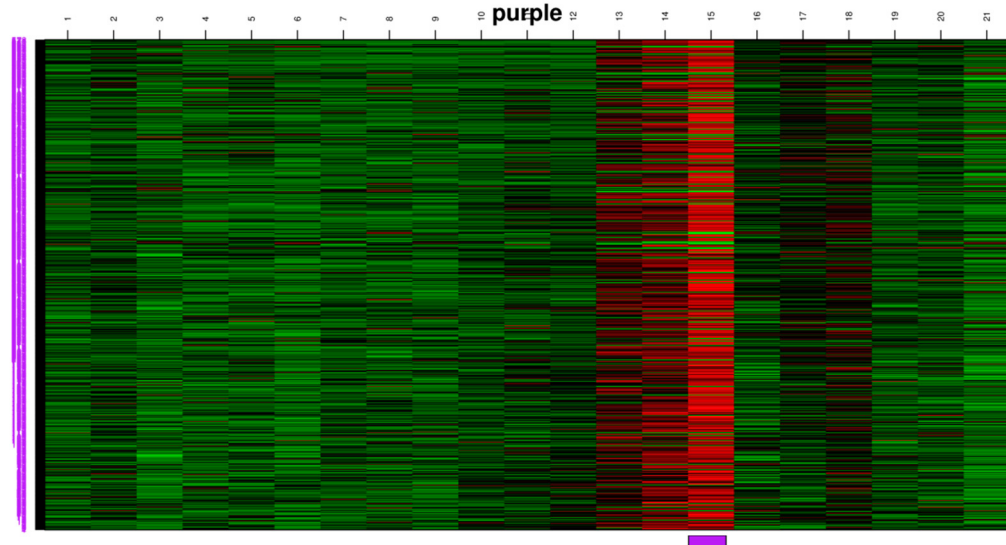

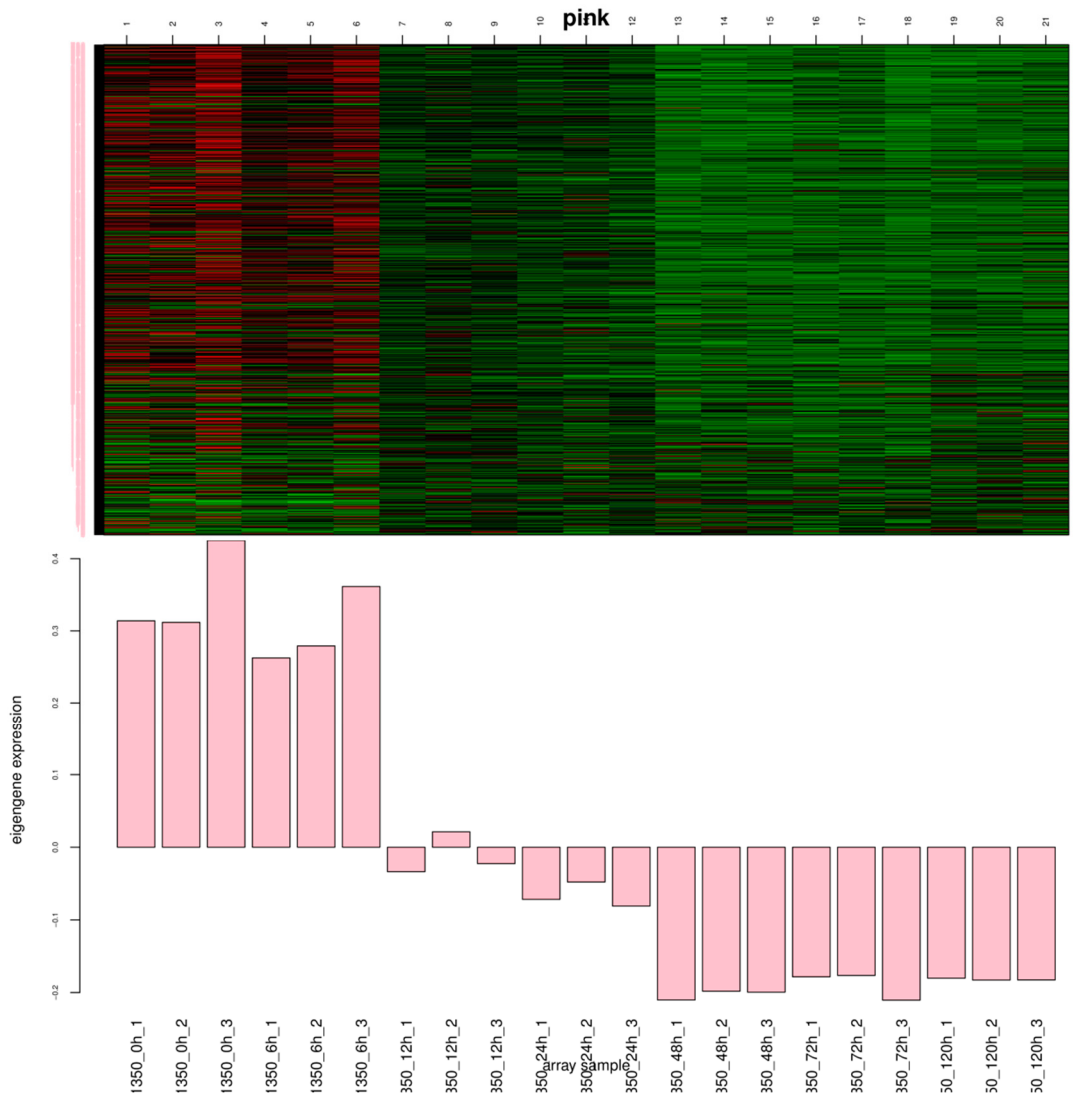

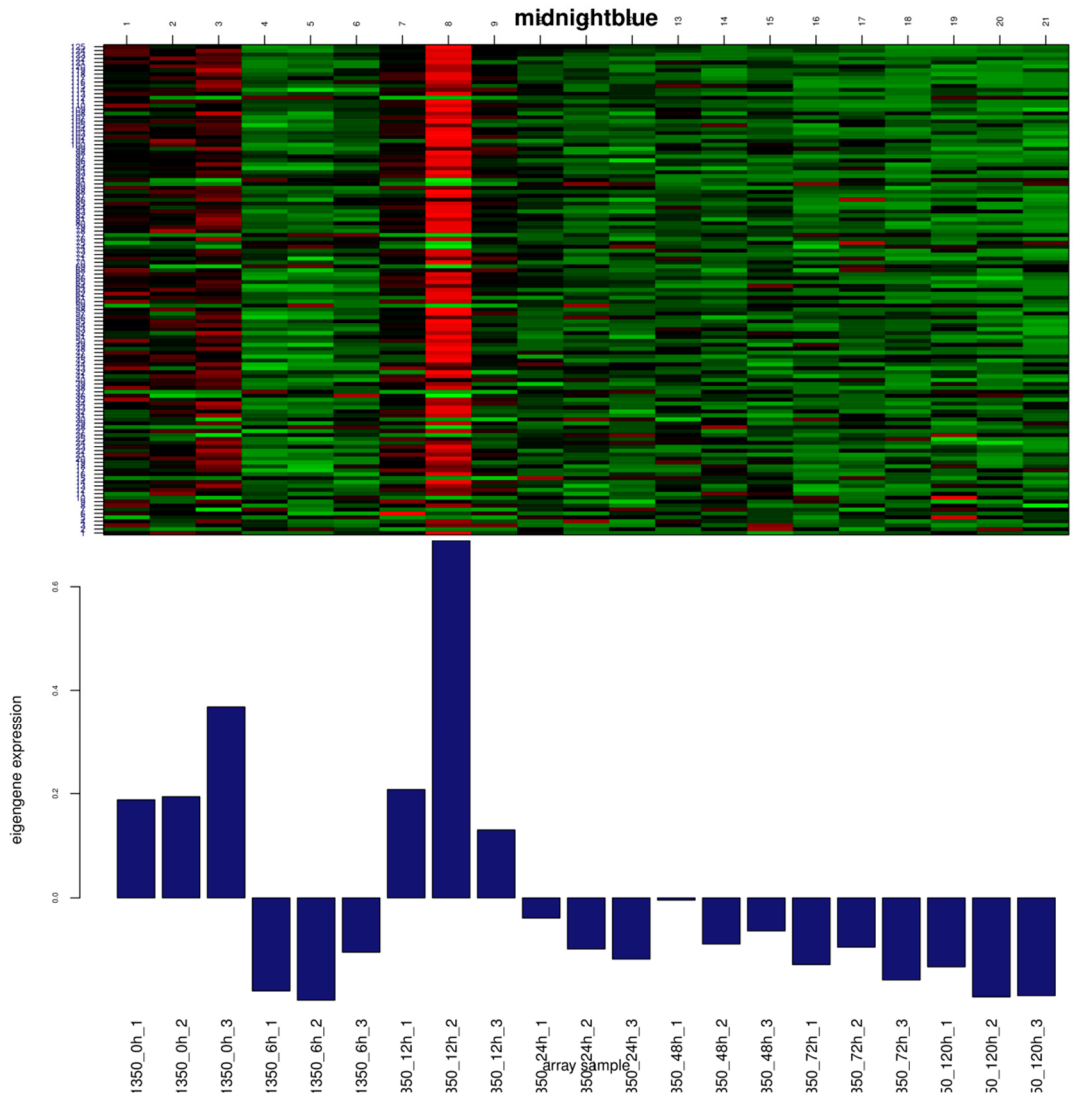

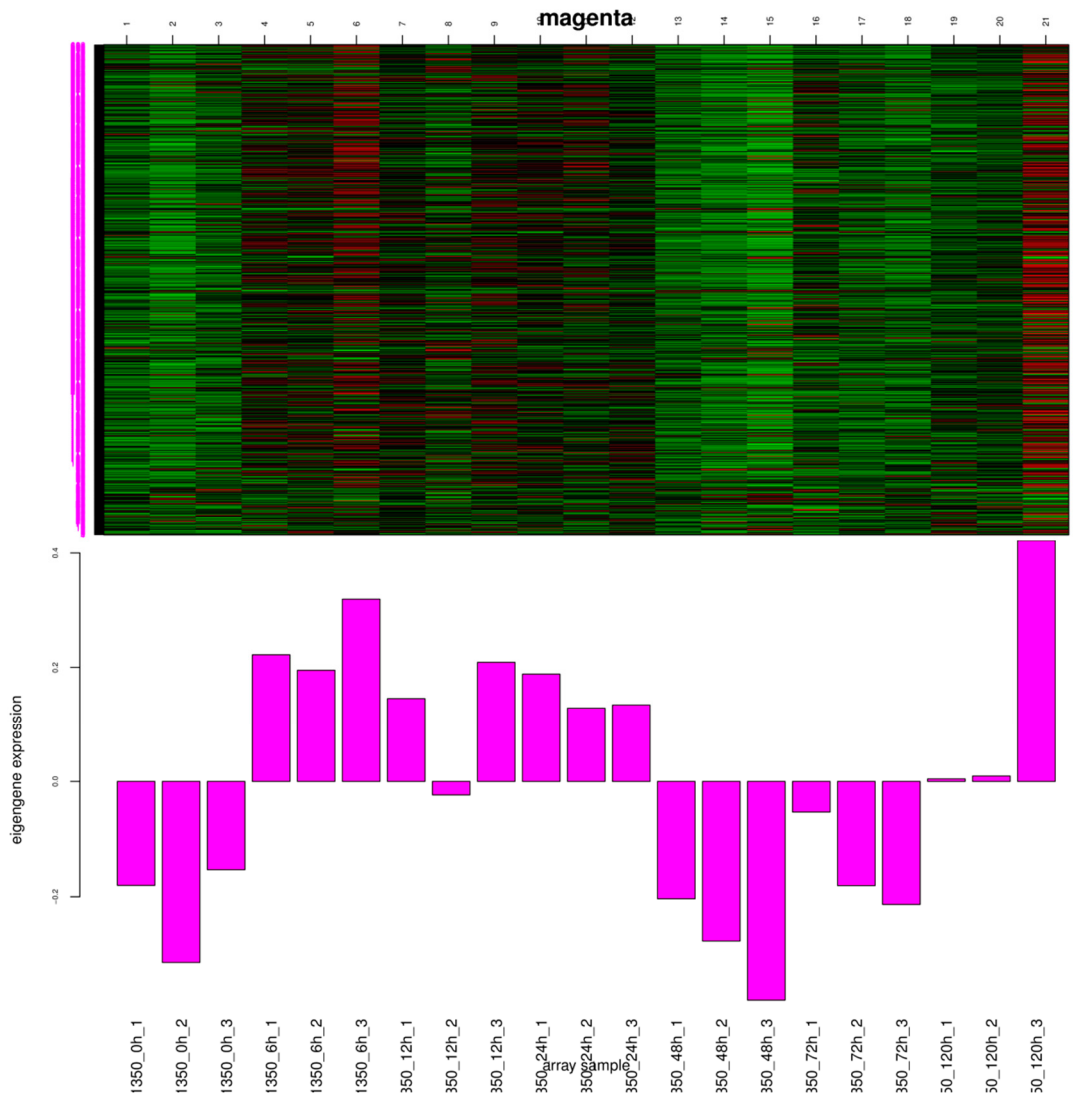

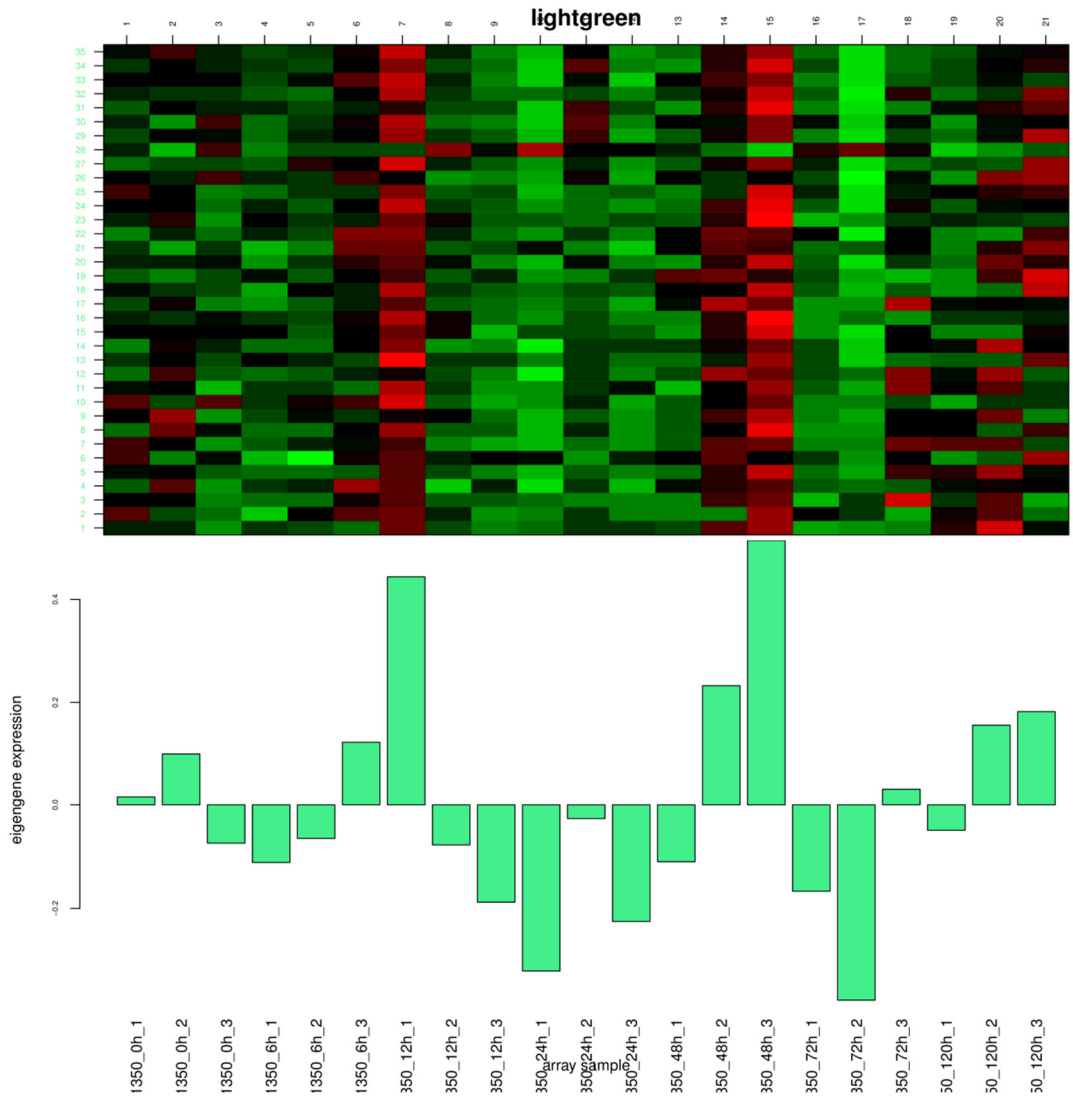

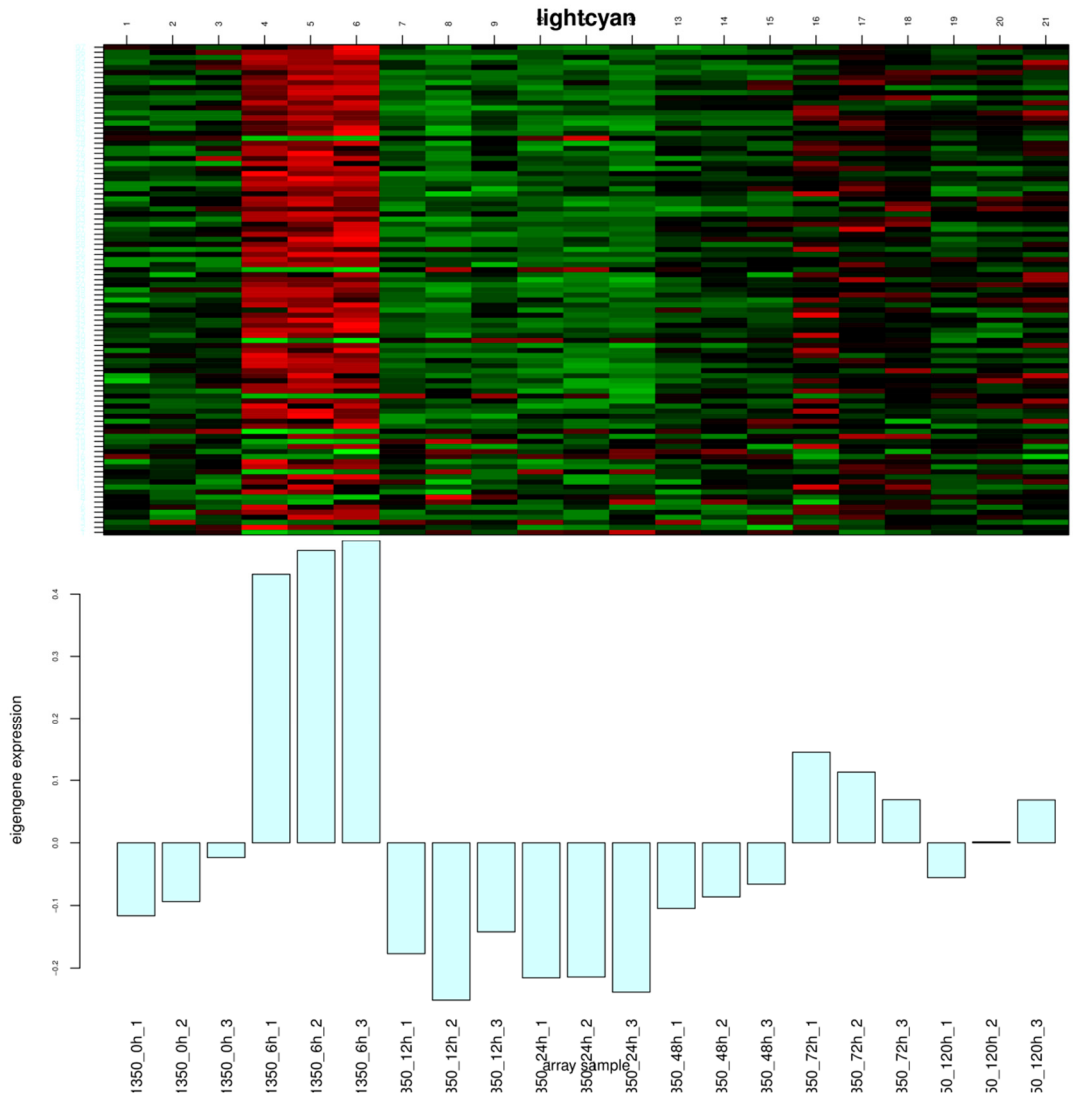

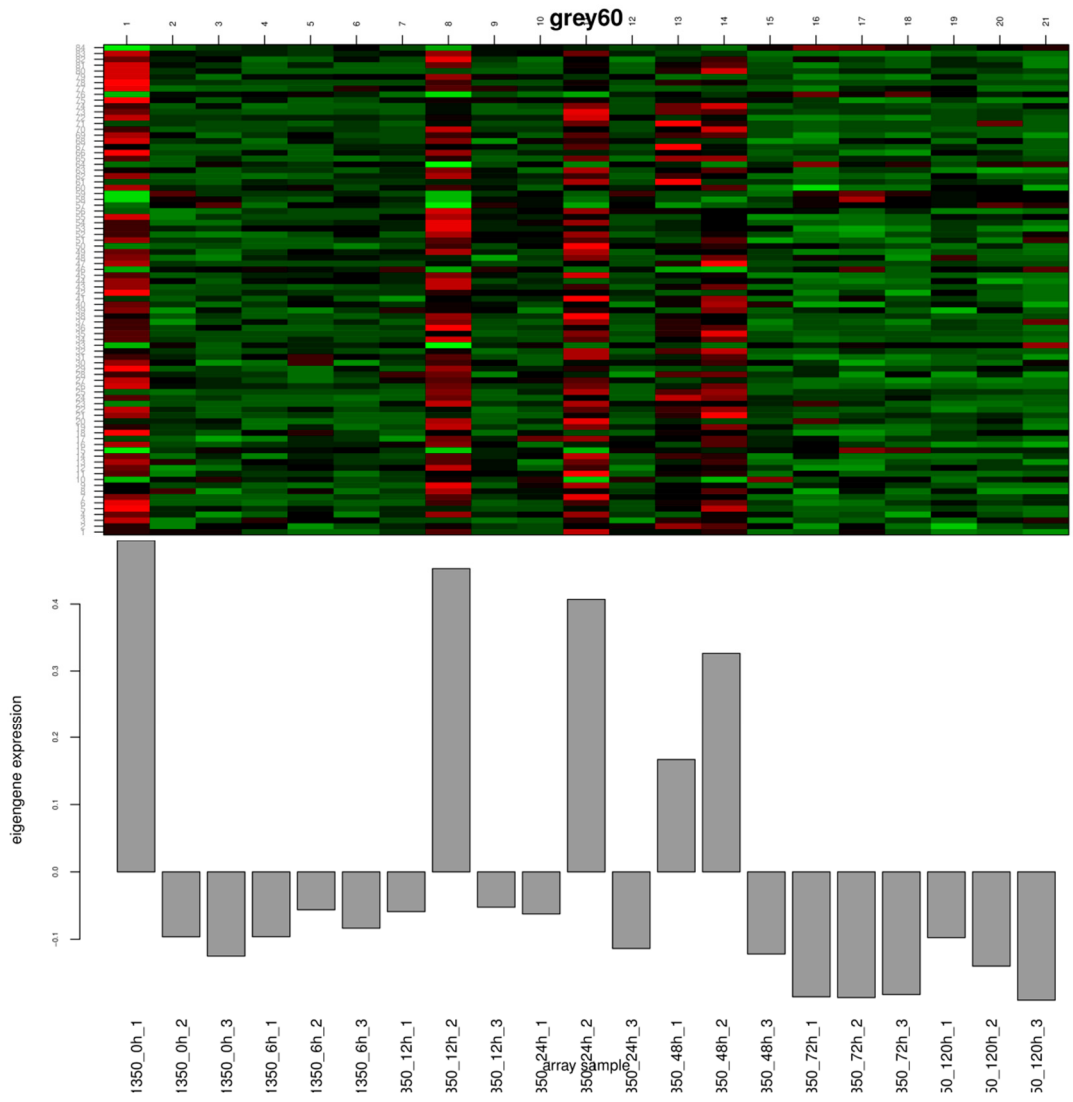

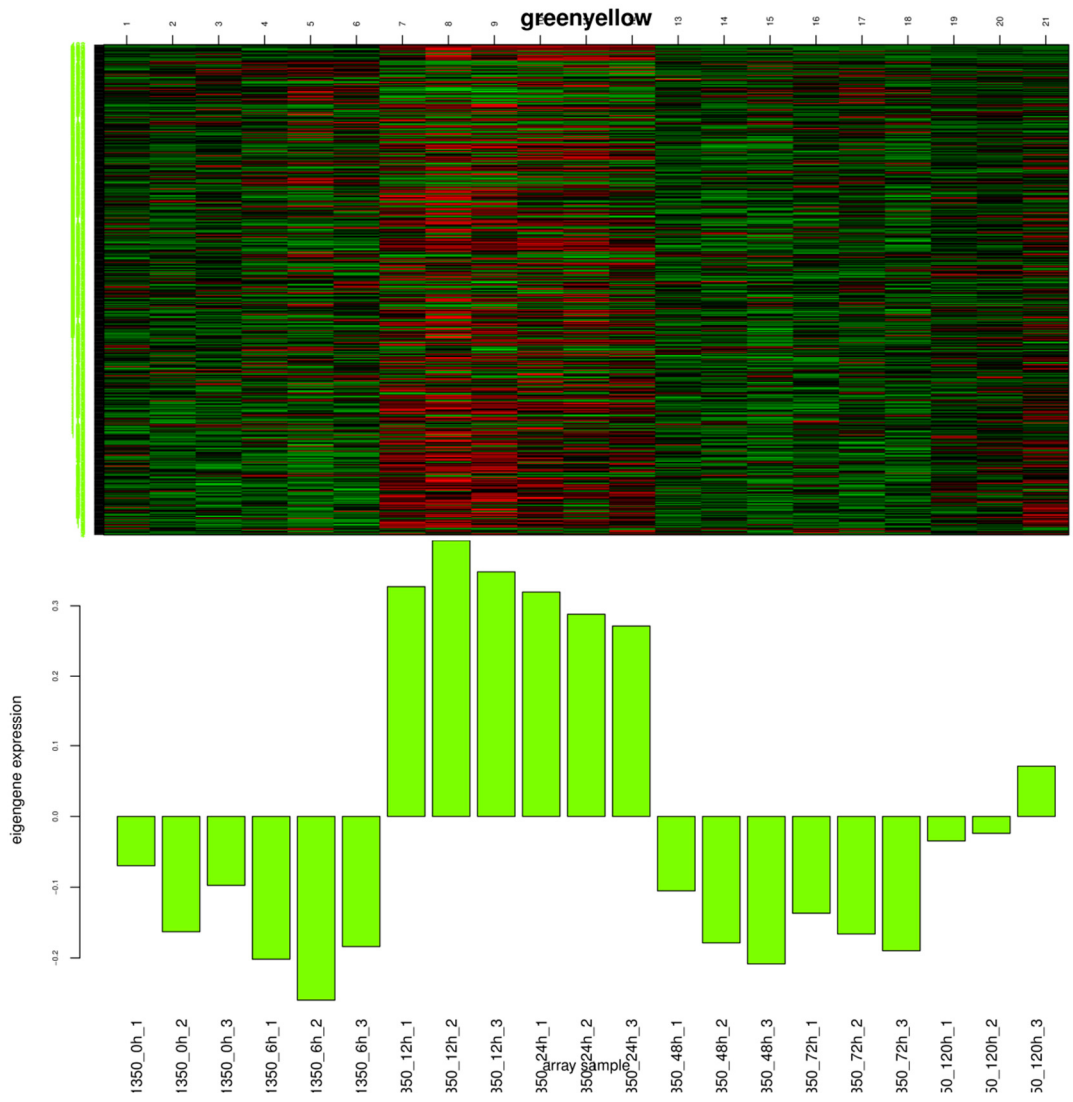

eigengene expression

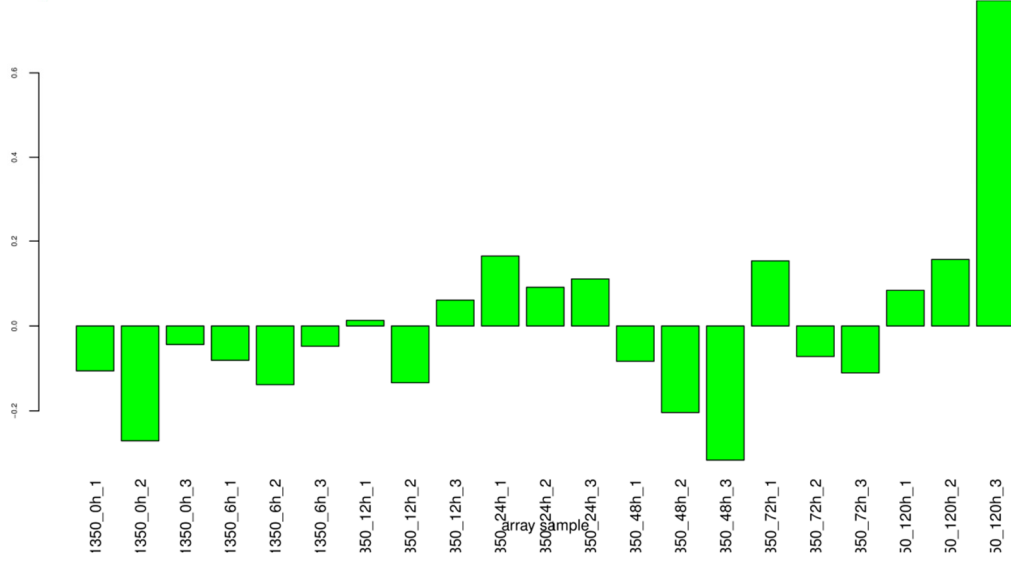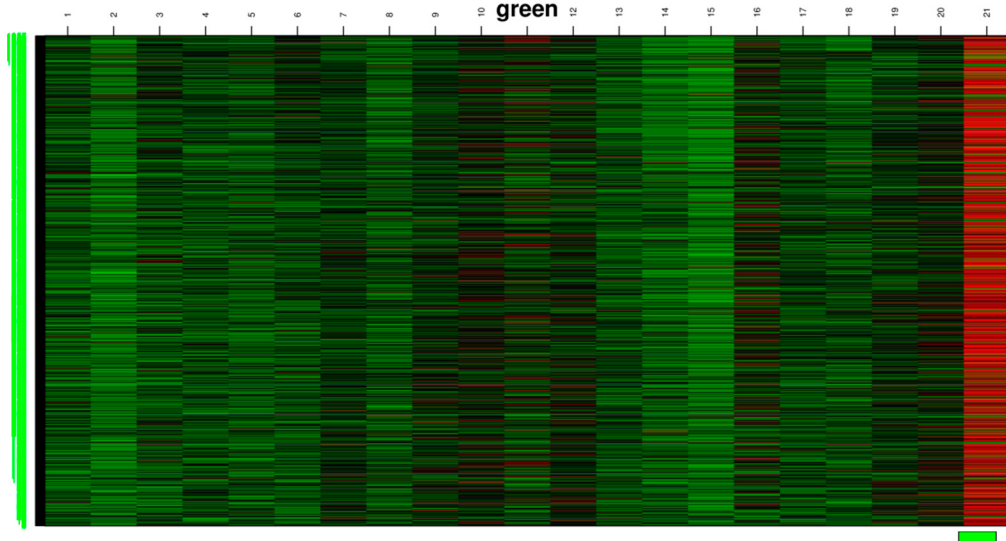

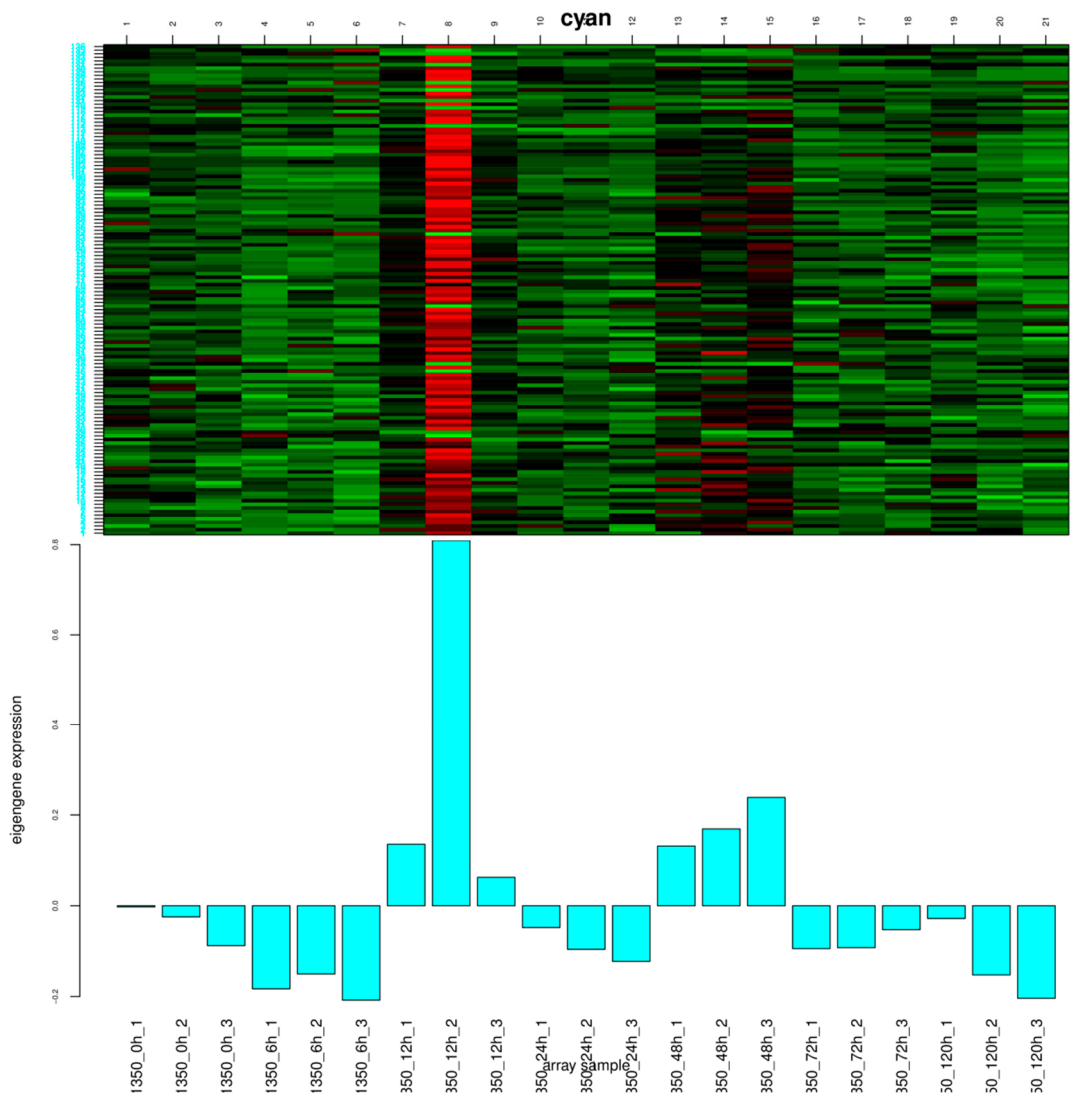

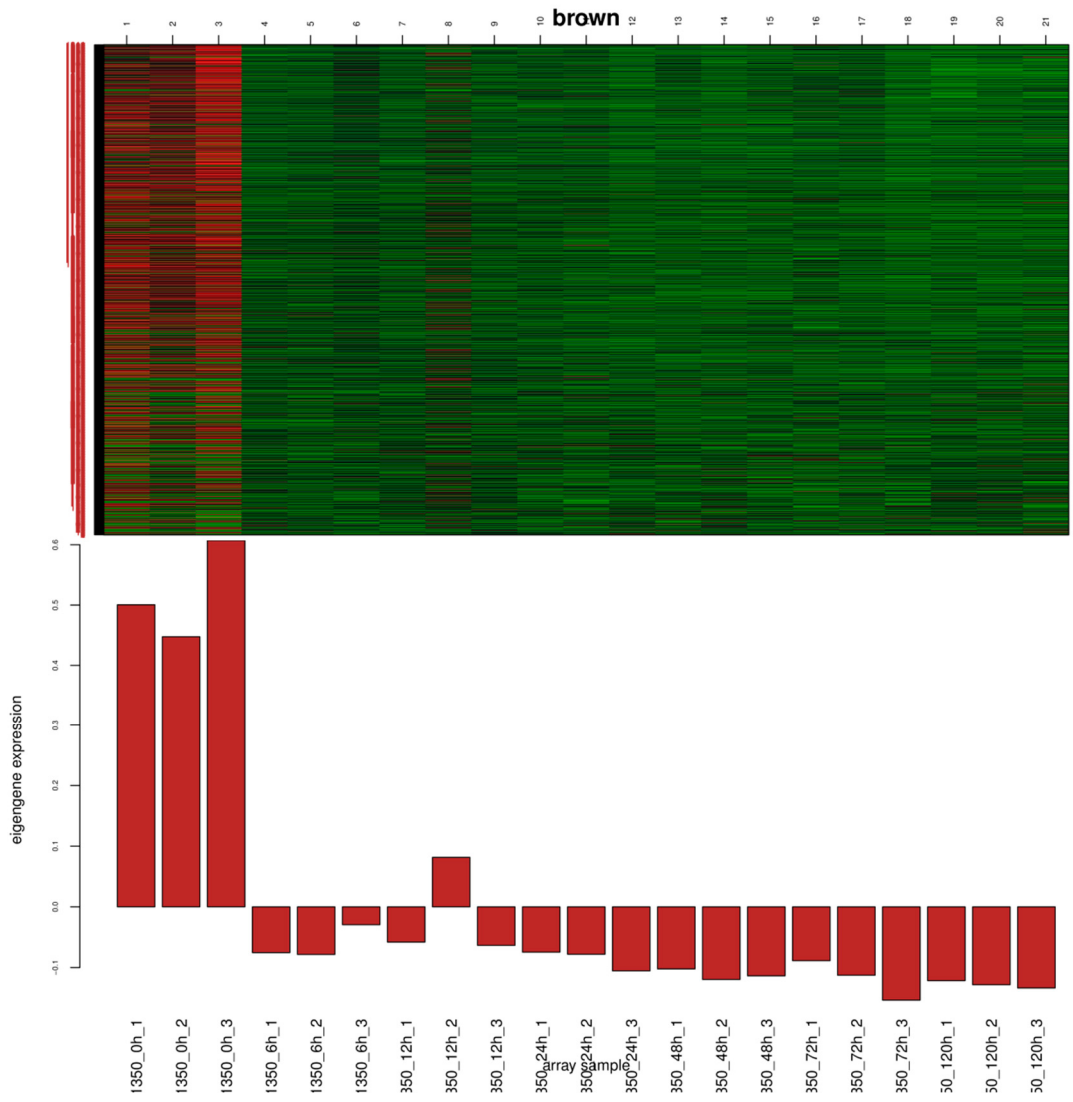

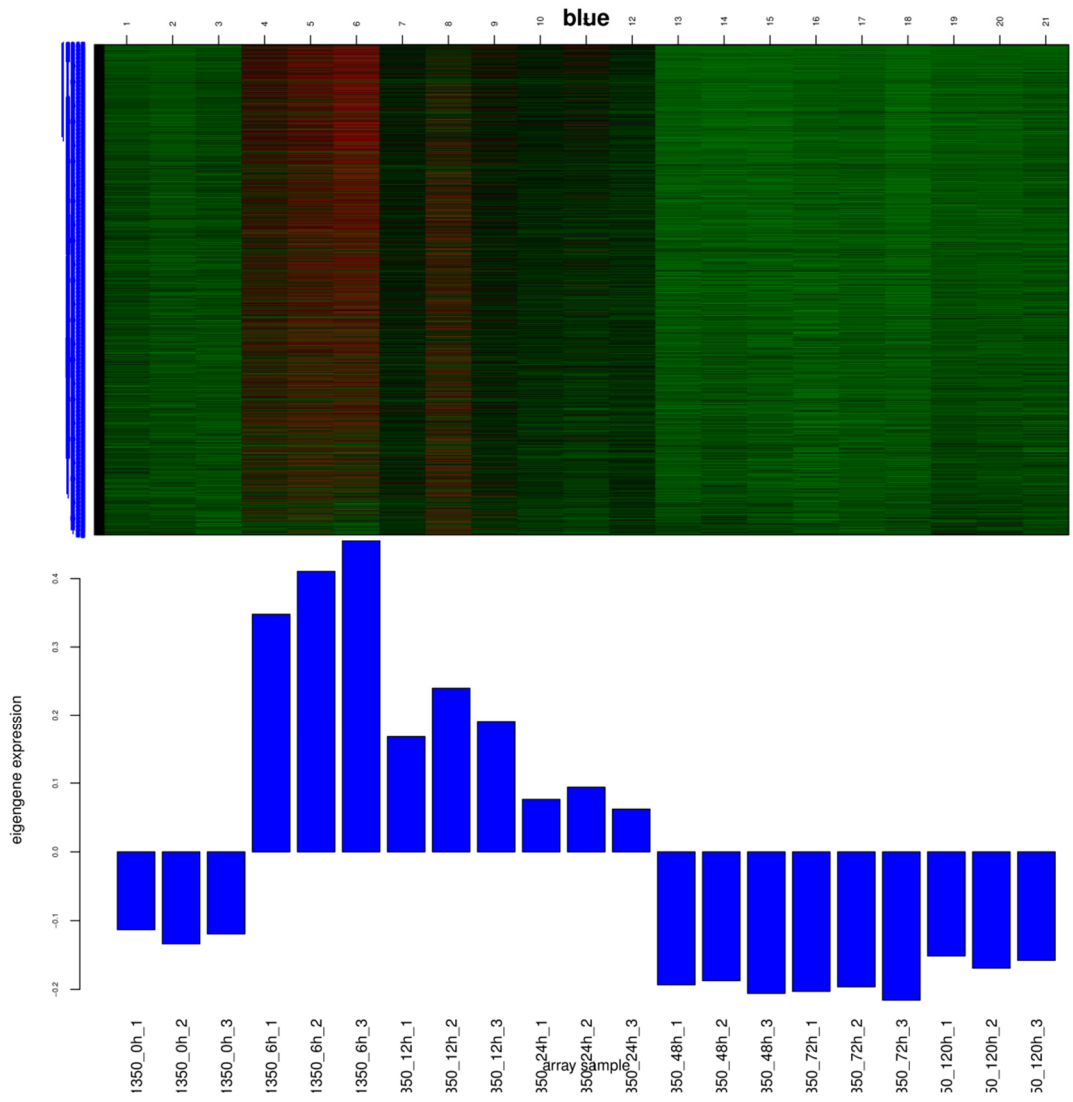

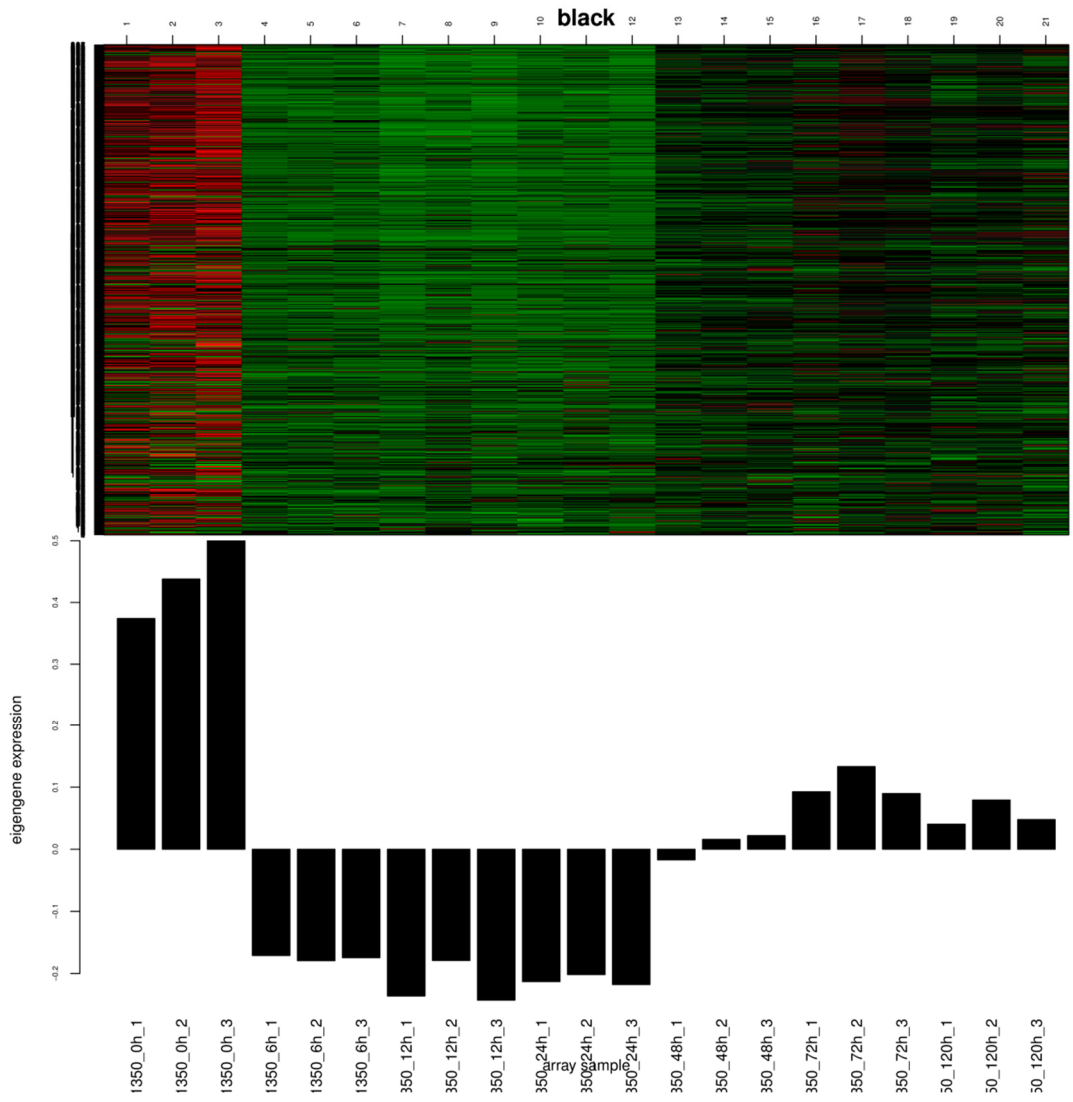

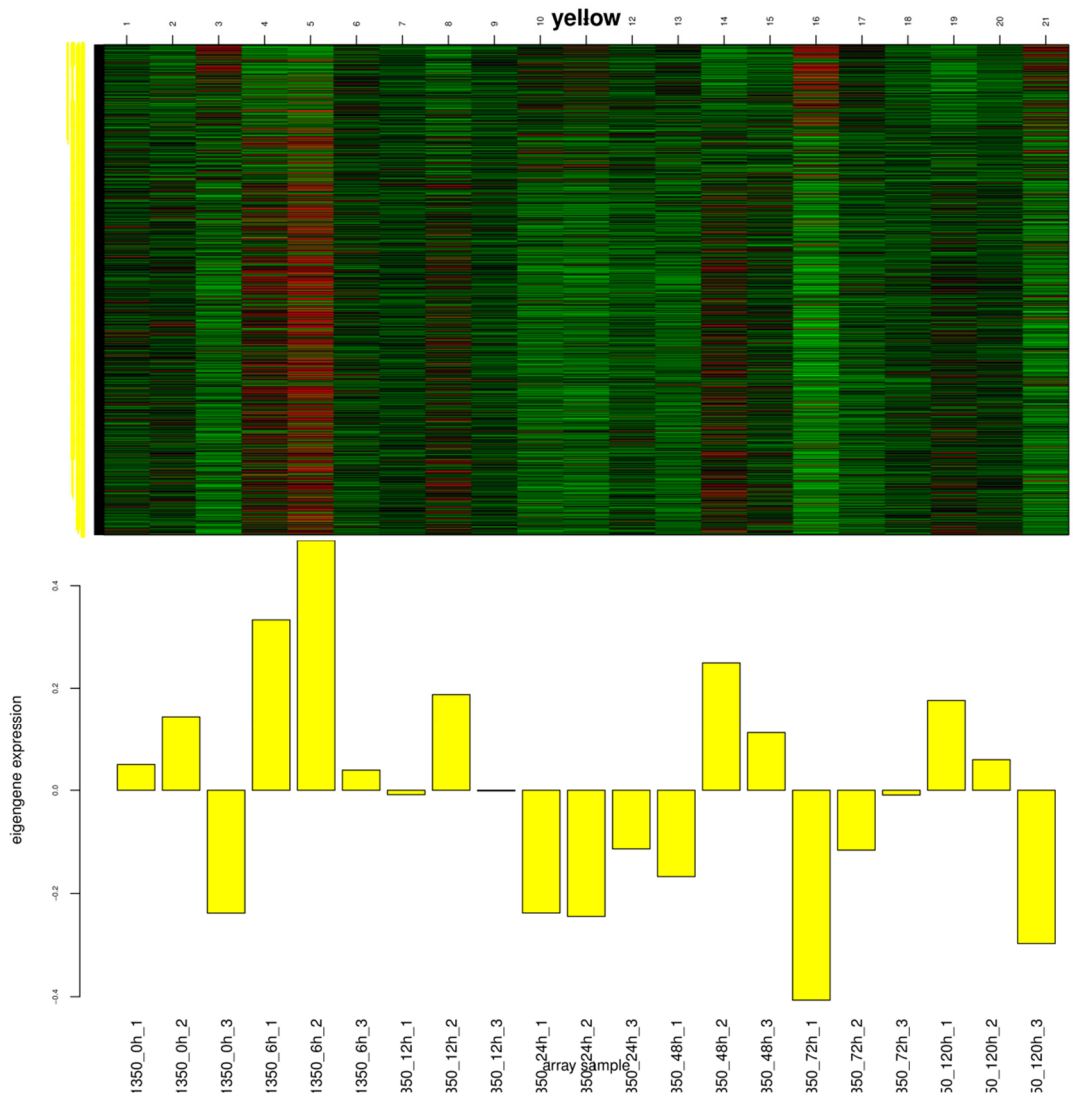

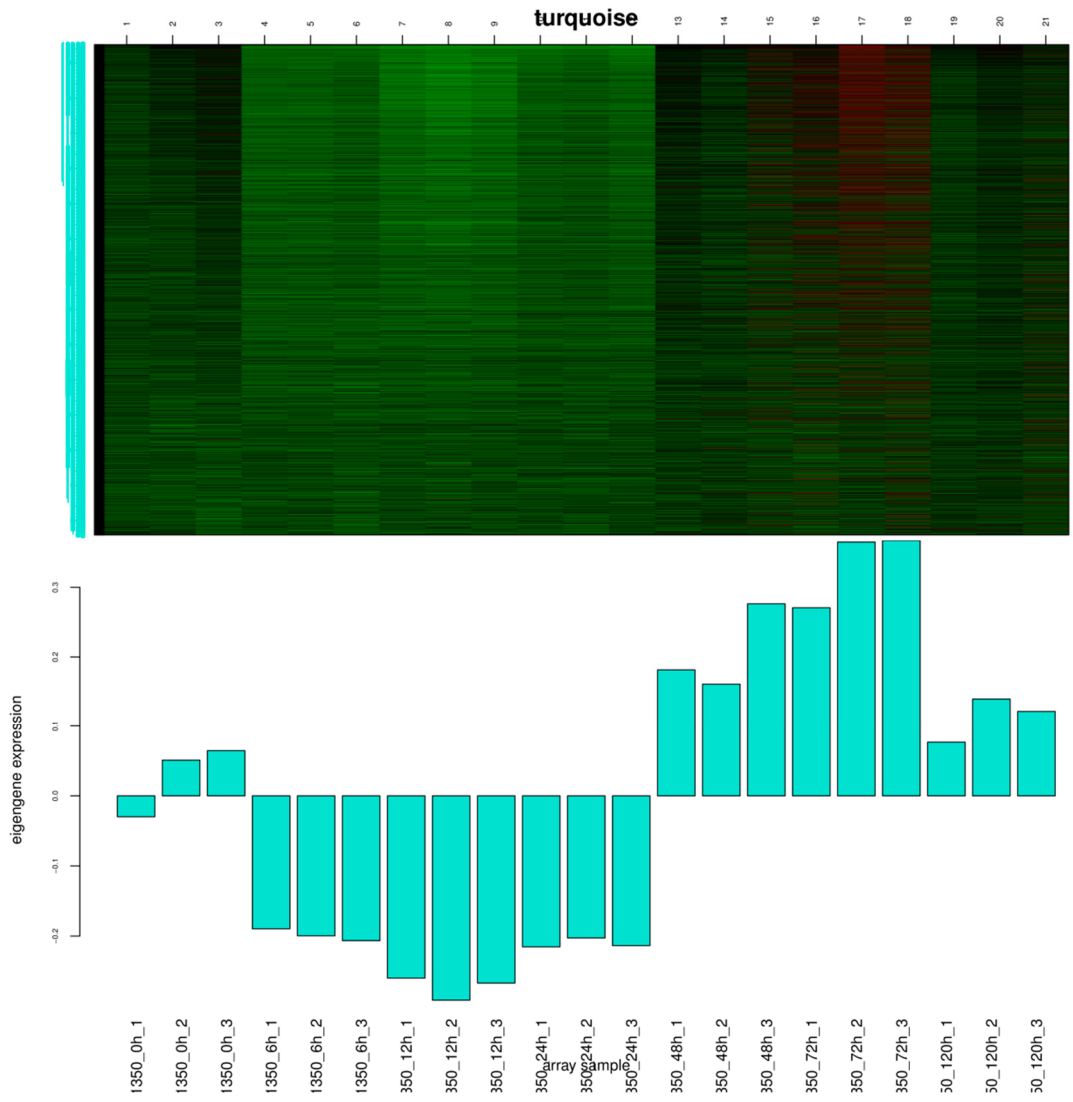

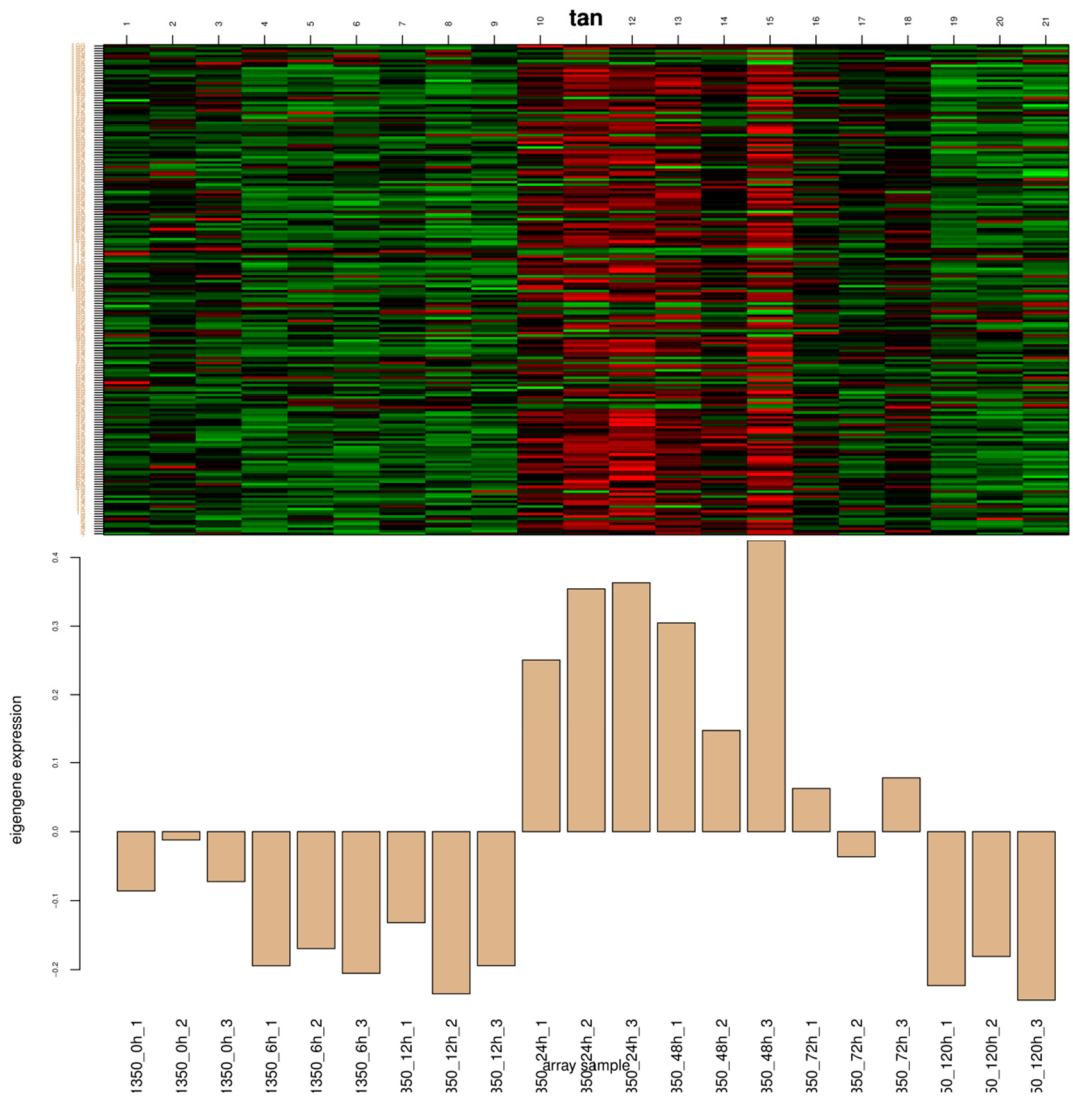

Supplement: Supplementary file 1 [file ijms-26-05538-s001.zip › Heatmap.pdf]
